# Diversity of House Dust Mite Species in Xishuangbanna Dai, a Tropical Rainforest Region in Southwest China

**DOI:** 10.1155/2015/421716

**Published:** 2015-05-04

**Authors:** Jing-Miao Yu, Qing-Hua Luo, Jin-Lu Sun, Cun-Lian Shi, Jia Yin, Yu-Ling Zhou, Rui Tang, Hui Zhang, Zhang Yu, Meng Chen

**Affiliations:** ^1^Department of Allergy, Peking Union Medical College Hospital, Chinese Academy of Medical Sciences and Peking Union Medical College, Beijing 100730, China; ^2^Department of Dermatology and Venereology, Xishuangbanna Dai Autonomous Prefecture People's Hospital, Yunnan 666100, China

## Abstract

*Purpose*. To survey the species diversity of home dust mites (HDM) in Xishuangbanna, a tropical rainforest region in Southwest China.* Methods*. From August 2010 to January 2011, mite-allergic patients and healthy controls were invited to participate. Dust samples from the patients' homes were collected, and mites in the samples were isolated. Permanent slides were prepared for morphologically based species determination. *Results*. In total, 6316 mite specimens of morphologically identifiable species were found in 233 dust samples taken from 41 homes. The result shows that the mite family of Pyroglyphidae occupied the highest percentage of the total amount of mites collected, followed by Cheyletidae family. The most common adult Pyroglyphidae mites were * Dermatophagoides* (*D.*) *farinae*, *D. pteronyssinus*, and *D. siboney*. The most common mites found from other families were *Blomia tropicalis*, *Tyrophagus putrescentiae*, and *Aleuroglyphus ovatus*. Four main allergenic dust mite species *D. farinae*, *D. pteronyssinus*, *D. siboney*, and *Blomia tropicalis* were found to be coinhabiting in 6/41 homes. *Conclusion*. The HDM population in homes in Xishuangbanna, a tropical rainforest region in Southwest China, has its own characteristics. It has rich dust mite species and the dust mite densities do not show significant variation across seasons.

## 1. Introduction

Presently, approximately 10 million Chinese children suffer from asthma and about 50 million Chinese adults have allergic rhinitis [1–6]. In the past ten years, the allergic diseases have increased in prevalence all over the world and have become a public health problem [5–7]. Various kinds of allergens can lead to allergic diseases. Among these allergens, home dust mites have been considered as the major source of allergen and more than 50% of allergic diseases in clinics are attributed to them [8]. Therefore, research of HDM region distribution may help in allergic diseases' prevention and treatment [9].

China is large, and its climate varies from tropical zone to cold temperate zone. It is evident that the predominant allergenic mite species differ across diverse regions of China [10–13]. For example, in Northern China region (Beijing), the predominant species were* D. farinae*,* D. pteronyssinus*, and* D. siboney*. In Central China region (Shanghai), the predominant species were* D. pteronyssinus*,* Hirstia domicola*, and* Glycyphagus privatus*. In Southern China region (Guangzhou), the predominant species were* D. pteronyssinus*,* D. farinae*, and* B. freemani*. In Guangxi province, a subtropical region in Southern China, the predominant species were* D. farina* and* D. pteronyssinus*. These surveys of the distribution of domestic mites in China have been conducted mostly in the densely populated and highly industrialized central cities of China. Wide use of air-conditioner in industrialized cities may be related to growth of dust mites.

There are no reports about HDM surveys in tropical rainforest area of China. In the present study, to improve our understanding of the national epidemic of allergic diseases in China, we investigated the distribution of HDM in Xishuangbanna Dai, P.R. China, which is a special city in a tropical rainforest biome. We compared mite distributions in Xishuangbanna area homes between its rainy and dry seasons and compared the seasonal distributions in Xishuangbanna and in Beijing [10, 11].

## 2. Materials and Methods

### 2.1. Study Groups and Allergy Tests

The survey subjects included a case group and a control group. The case group consisted of 31 homes of patients who were admitted to Xishuangbanna Dai Autonomous Prefecture Hospital and diagnosed by specialists with mite-related allergic diseases between August 2010 and January 2011. Their diagnoses were based on comprehensive considerations about disease history, symptoms, signs, skin prick test results, and serum sIgE test results. The case group only included homes where patients had positive skin prick test results from* D. farinae* and* D. pteronyssinus* (ALK Denmark skin prick test solution) and sIgE positivity of at least 2 classes (d1 and d2 from Phadia, Sweden). The control group consisted of 10 homes without any mite-allergic patients selected via the same diagnostic procedure. In accordance with Good Clinical Practice, we acquired their permission to collect their house dust. The patient information was anonymized. The Institutional Review Board of Peking Union Medical College Hospital approved the study protocol.

### 2.2. Dust Collection Methods

Dust samples were collected with a 1200 W vacuum attached securely to an ALK Dust Trap (ALK. Copenhagen, Denmark) with a vacuum hose and O-ring and a measuring device to define 1 m^2^ of collection area or a scale. The filter dish was removed carefully to prevent dust spillage. Each collection dish was covered with a lid, sealed, and labeled with date and place information. The vacuum nozzle was stored vertically to prevent dust from falling out after vacuuming. The nozzle was rinsed and dried before being fitted with a new filter dish for collection of the next sample.

Collection sites were locations where mites survive and breed easily (i.e., pillows, quilts, sheets, sleeping pads and mattresses, sofas, rugs, and carpet floors). Collections were made by placing the vacuum nozzle over a defined 1 m^2^ area for 3 minutes. Samples were collected from the entire selected surface. The collection time was shortened to 2 minutes for places with surface area less than 1 m^2^ (such as pillows, sofas, and rugs).

All dust samples were collected and prepared by two researchers (Qing-Hua Luo and Yu-Ling Zhou). For each sample, a record of which collection device and filter plate were used, the collection location, and other related information such as the family's living conditions was kept. Dust samples were transported to the lab in Xishuangbanna. Whenever possible, the mites within the samples were isolated immediately; otherwise, they were stored frozen at −20°C for later isolation [10, 11].

### 2.3. Isolation, Storage, and Identification of Mites

The flotation method was used to extract mite bodies from the dust samples. Isolated mite specimens were stored in 70% alcohol. For convenient morphological species identification of individual mites, permanent slides were prepared using Hoyer's Medium and observed under a light microscope. Species were identified in accordance with the morphological characteristics described by Krantz and Walter and other related information [10, 11, 14–16].

## 3. Data Analysis

The mite-positive rate of samples was calculated as follows: positive rate is positive sample number/total sample number × 100%. The number of mites in each mite species within each sample was counted, and the percentage of each species relative to the total mite counted for each sample was calculated. All mite bodies were counted, whether they were alive, dead, or physically damaged. Mite density was calculated for each sample as follows: mite density is total number of mites detected/weight of isolated dust (in g). If the number of mite species obtained from the same location at different time points differed, the average number was used for the distribution calculations. The Xishuangbanna HDM data obtained in this study were compared to analogous data from a study conducted in Beijing during the same time span from December 2008 to January 2010 (*n* = 38 homes) [10, 11].

Rank sum tests for two independent samples (Mann-Whitney *U* test) were used to compare mite density data between Xishuangbanna and Beijing. A Chi-square test of two independent samples was used to compare the prevalence of mites between Xishuangbanna and Beijing. A row mean score differ statistic was used to compare the dust mite densities between the case and control groups. Statistical analysis was conducted with statistical software SPSS 13.0. A statistical level of *α* = 0.05 was considered significant for two-tailed tests.

## 4. Results

### 4.1. Characterization of Dust Mite Population and Distribution

A total of 233 dust samples were collected from 41 homes in the Xishuangbanna area between August 2010 and February 2011. Mites were detected in 186/233 samples (79.8%) and 40/41 homes (97.5%). In total, 6,349 live, intact mites in various development stages were detected. They were distributed among 877 slides. Species identification was possible for 6316/6349 of the mites. This group of 6316 identified specimens (the damaged remains of dead mites were not identified) included mites spanning 23 species in 15 genera, representing 12 families belonging to 3 orders of Acari. Notably, the four main allergenic HDM including* D. farinae*,* D. pteronyssinus*,* D. siboney*, and* Blomia (B.) tropicalis* were found together, for the first time in China, in 6/41 homes. Additionally, 40 individual insects (class Insecta, order Psocoptera) were identified; they included two barklice species (family Liposcelididae) of the same genus (Table 1).

The numbers and percentages of individual arachnids and insects of particular species are reported in Table 1, together with the numbers and percentages of dust samples and homes positive for particular species. The Pyroglyphidae family was most prevalent among the mite species detected (96.6%), followed by Cheyletidae (2.0%). The small minority of non-Pyroglyphidae mites isolated were composed of Prostigmata (57.1%), Acaridae (16.3%), Oribatida (12.0%), other Astigmata (10.4%), and Mesostigmata (5.2%).

Among the predominant Pyroglyphidae mites, most were adults (85.7%), followed by nymphs (13.4%) and larvae (0.9%).* D. farinae* made up more than three quarters (77.0%) of the adult Pyroglyphidae mites (Table 1), including 1483/4001 (37.1%) males and 2518/4001 (62.9%) females.* D. pteronyssinus*, the second most prevalent Pyroglyphidae, constituted about a fifth of the adult Pyroglyphidae mites (21.6%), including 511/1128 (45.3%) males and 617/1128 (54.7%) females.* D. siboney* ranked a distant third (1.9%), including 49/101 (48.5%) males and 52/101 (51.5%) females.

The detailed counts and percentages of mites of the particular species of mites observed are reported in Table 1. Briefly, the order of mite specimen prevalence overall was* D. farinae* ≫* D. pteronyssinus* ≫* D. siboney* >* Tyrophagus putrescentiae* >* B. tropicalis > Aleuroglyphus ovatus*. The distribution of homes positive for each of these species followed a similar order, except that* T. putrescentiae* and* B. tropicalis* were found in an equal number of homes. Hence, although most of the aforementioned species were present in low numbers, relative to* D. farinae*, they were widespread in Xishuangbanna area homes.

Multiple mite species were often found within homes and within dust samples. Among the 41 surveyed homes, 2 homes had eight species of mites, 1 had seven species, 2 had six species, 6 had five species, 14 had four species, 11 had three species, 3 had two species, 1 had one species, and only 1 home had no mites. The predominant mite species was* D. farinae* in most of the homes (33/41; 80.5%) and* D. pteronyssinus* in about one-sixth of the homes (7/41; 17.1%). In homes with duplicate collection, the predominant mite species did not change. Generally, the predominant mite species within each sample constituted greater than 70% of the mites observed in that sample, and species predominance was consistent across samples from homes that were sampled more than once.

### 4.2. Mite Density Comparisons between Seasons and Regions

**Figure 1:**
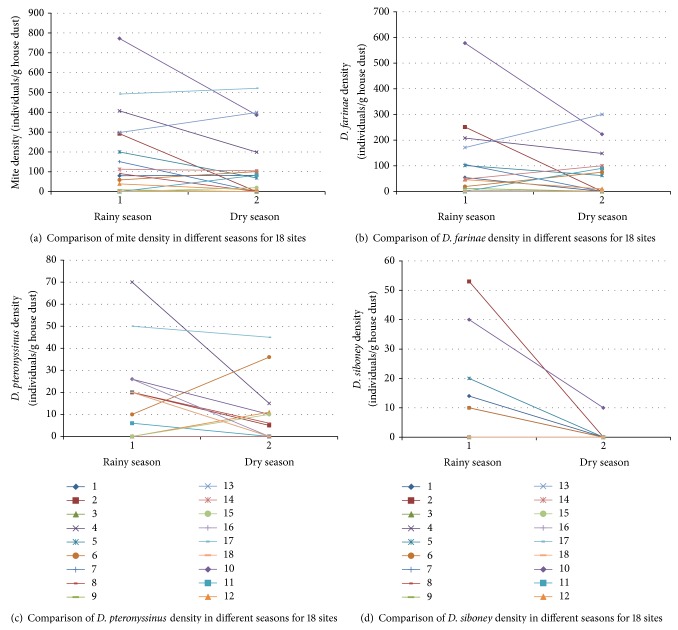
Comparisons across the seasons of total mite (a),* D. farinae* (b),* D. pteronyssinus *(c), and* D. siboney* (d) densities at 18 sites.

Domestic mite densities in Xishuangbanna area are shown by season in Figures 1(a)–1(d). We found the mean mite density for 18 sites was 171.7 mites/g in wet season and 111.1 mites/g in dry season, respectively (Figure 1(a)). Although the mean mite density in wet season was higher than that in the dry season, this difference was not significant (*P* = 0.103 > 0.05) after Wilcoxon signed rank test by two paired samples for these two seasons. Also, we found there were no statistical differences in mite density for both* D. farinae* (Figure 1(b)) and* D. pteronyssinus* (Figure 1(c)) after paired *t*-test between wet season and dry season (*P* = 0.311 > 0.05 for* D. farinae* and *P* = 0.091 > 0.05 for* D. pteronyssinus*). In addition, the mite density for* B. tropicalis* in wet season was higher than that in dry season (*P* = 0.02 < 0.05) (Figure 1(d)).

The mite densities and positivity rates in the Xishuangbanna area differed markedly from analogous data for samples collected in the Beijing area (December 2008 to January 2010) [4, 5], revealing a significant geographical variation in mite populations (*P* < 0.001). The positive detection rate (positive sample number/total sample number) for domestic mites was higher in Xishuangbanna (79.8%) than in Beijing (64.6%; *P* < 0.001).

### 4.3. Domestic Mite Density in Case versus Control Groups

As reported in detail in Table 2, we found no differences in mite densities between the case group (homes of patients with mite allergies) and the control group (homes of healthy controls) (*P* = 0.2697 > 0.05). The two groups had similar representations of low-density (≤100 mites per g of dust), medium-density (≤100 mites per g of dust), and high-density (>500 mites per g of dust) samples, as well as similar densities overall (Table 2).

## 5. Discussion

The present results confirm that home dust mites exist in Xishuangbanna area. We found that* D. farinae* was the most prevalent mite species in Xishuangbanna home dust samples, as is the case in Beijing [10, 11]. In contrast, the most prevalent mite species observed in Taiwan [17] and Hong Kong [18], cities which have latitudes similar to Xishuangbanna area, were* D. pteronyssinus* and* B. tropicalis*, respectively. This difference may be due to the difference of the environment and climate. For example, Xishuangbanna is inland and tropical, whereas Taiwan and Hong Kong are coastal and subtropical. Interestingly, a similar dissociation of cites at similar latitudes was found between Italy and the USA, where* D. farinae* and* D. pteronyssinus* were found to be the predominant mite species, respectively [19, 20].

The factors affecting the distribution and abundance of mite species are quite complex, involving not only geographical factors such as latitude, seasonality, climate, rainfall, altitude, and distance from a coast, but also household factors such as neighborhood location, building age and materials, house orientation, ventilation and thermal systems, family economic conditions, surrounding foliage, types of furnishings, and number of occupants and their smoking status and health habits [21–24]. It is clear that the concentration of mite allergens in houses is influenced by multiple factors related to climate, housing design, and the behavior of the occupants. In the current study, for the domestic mite density, there was little seasonal effect and no difference between the houses of mite-allergic patients with asthma or allergic rhinitis and those of healthy controls. This may be caused by the special climate of the region. In a consistently humid and warm climate, mite exposure is likely to be present in all houses.

*D. siboney* and* B. tropicalis*, which were also detected in this survey, albeit at lower amounts, are major allergen culprits in some subtropical and tropical regions. For example,* D. siboney* is the predominant mite allergen in Cuba [14] and Puerto Rico [15], whereas* B. tropicalis* is the predominant mite allergen in Singapore [25], Malaysia [26], the Philippines [27], Taiwan [17], and Hong Kong [18]. Although* D. pteronyssinus, D. siboney*, and* B. tropicalis* were minor species in Xishuangbanna in terms of percentage of dust mites observed, relative to* D. farinae*, they were detected commonly. Indeed, for the first time,* D. farinae, D. pteronyssinus, D. siboney*, and* B. tropicalis* were found to be coinhabiting. Further study is needed to elucidate the importance of these minor mite species in allergic disease in Xishuangbanna and other tropical regions.

Mite densities and positive detection rates were significantly lower in Beijing than in Xishuangbanna, perhaps due to their different geographical and climate characteristics and the large difference in latitude between the two areas. Indeed, it is not surprising that dust mites would be present in large numbers in tropical areas, such as Xishuangbanna, with mild temperature changes and a high relative humidity, which are optimal conditions for growth of the dust mites.

We observed no differences between the homes of mite-allergic people (case group) and homes of nonallergic people (control group) in terms of overall mite densities, nor in terms of numbers of homes with low, medium, or high densities of mites. Experts at an international seminar regarding mites and asthma held in 1988 reached a consensus that mite density of more than 100 mites per g of dust is a risk factor for sensitization and development of asthma (our cut-off between low- and medium-density samples) and that a mite density of more than 500 mites per g of dust is a risk factor for development of acute asthma attacks in mite-allergic patients (our cut-off between medium- and high-density samples). These cut-off values were used in our case versus control comparisons because they have been accepted indicators in mite research internationally for the last two decades [28]. The fact that there were no differences between mite densities in the case versus control groups underscores the notion that mites are free-living organisms whose living patterns are independent of human beings, while also arguing against the perspective that allergic patients' symptoms are due to higher-than-average mite levels in their homes. We consider that the allergic diseases are associated with both of the complex environmental elements and the individual genetic characteristics rather than a single factor such as the home dust mite density.

In conclusion, the present study showed that the HDM population in Xishuangbanna area has its own characteristics. It has rich dust mite species. Four main allergenic dust mite species including* D. farinae, D. pteronyssinus, D. siboney*, and* Blomia tropicalis* were found to be coinhabiting. Compared to Beijing area, the dust mite densities in Xishuangbanna area did not show significant variation across seasons. Primary investigation showed that there were no differences in mite densities between the homes of mite allergies and the healthy controls in this area.

## Figures and Tables

**Table 1:** Composition of mites and insects in house dust in Xishuangbanna area.

Species	Sampled homes^a,b^ (*n* = 41)		Samples^b^ (*n* = 233)		Mites^c^ (*n* = 6349)	
	Number	%	Number	%	Number	%
Acari	40	97.56	186	79.83	6316	99.48
Astigmata	40	97.56	183	78.54	6157	96.98
Pyroglyphidae	40	97.56	182	78.11	6102	96.11
*D. farinae *	40	97.56	167	71.67	4001	63.02
*D. pteronyssinus *	38	92.68	126	54.08	1128	17.77
*D. siboney *	20	48.78	39	16.74	101	1.59
Nymph	38	92.68	126	54.08	818	12.88
Larva	19	46.34	27	11.59	54	0.85
Acaridae	14	34.15	19	8.15	34	0.54
*T*.* putrescentiae *	10	24.39	16	6.87	28	0.44
*A*. *ovatus *	3	7.32	3	1.29	6	0.09
Glycyphagidae	13	31.71	16	6.87	19	0.30
*B. tropicalis *	10	24.39	16	6.87	19	0.30
Histiostomatidae	2	4.88	2	0.86	2	0.03
Oribatida	5	12.20	8	3.43	26	0.41
Haplochthoniidae	5	12.20	8	3.43	25	0.39
Unidentified	1	2.44	1	0.43	1	0.02
Prostigmata	27	65.85	54	23.18	123	1.94
Cheyletidae	27	65.85	54	23.18	123	1.94
Mesostigmata	5	12.20	8	3.43	10	0.16
Blattisociidae	3	7.32	6	2.58	8	0.13
Laelapidae Berlese	2	4.88	2	0.86	2	0.03

Insecta	10	24.39	12	5.15	33	0.52
Liposcelididae	6	14.63	6	2.58	22	0.35
Unidentified	6	14.63	7	3.00	11	0.17

Total number identified	40	97.56	186	79.83	6349	
Total number collected	41		233			

^a^Homes with more than one sample were regarded as the same home. ^b^The percentages of homes and samples were calculated with total number collected as the denominator. ^c^The percentage of individual specimens was calculated with total number identified as the denominator.

**Table 2:** Comparisons of the mite density in case homes versus control homes.

Group (number of samples)	Mite density, number of samples (%)		
	Low^a^	Medium^b^	High^c^
Case (*n* = 192)	110 (57.3%)	59 (30.7%)	23 (12.0%)
Control (*n* = 41)	21 (51.2%)	12 (29.3%)	8 (19.5%)

^a^≤100 mites/g dust. ^b^100–500 mites/g dust. ^c^>500 mites/g dust.

Note: the value of row mean score differ test was 1.2184 and the *P* value was 0.2697, which indicated that there were no differences in the mite densities between case and control homes.
